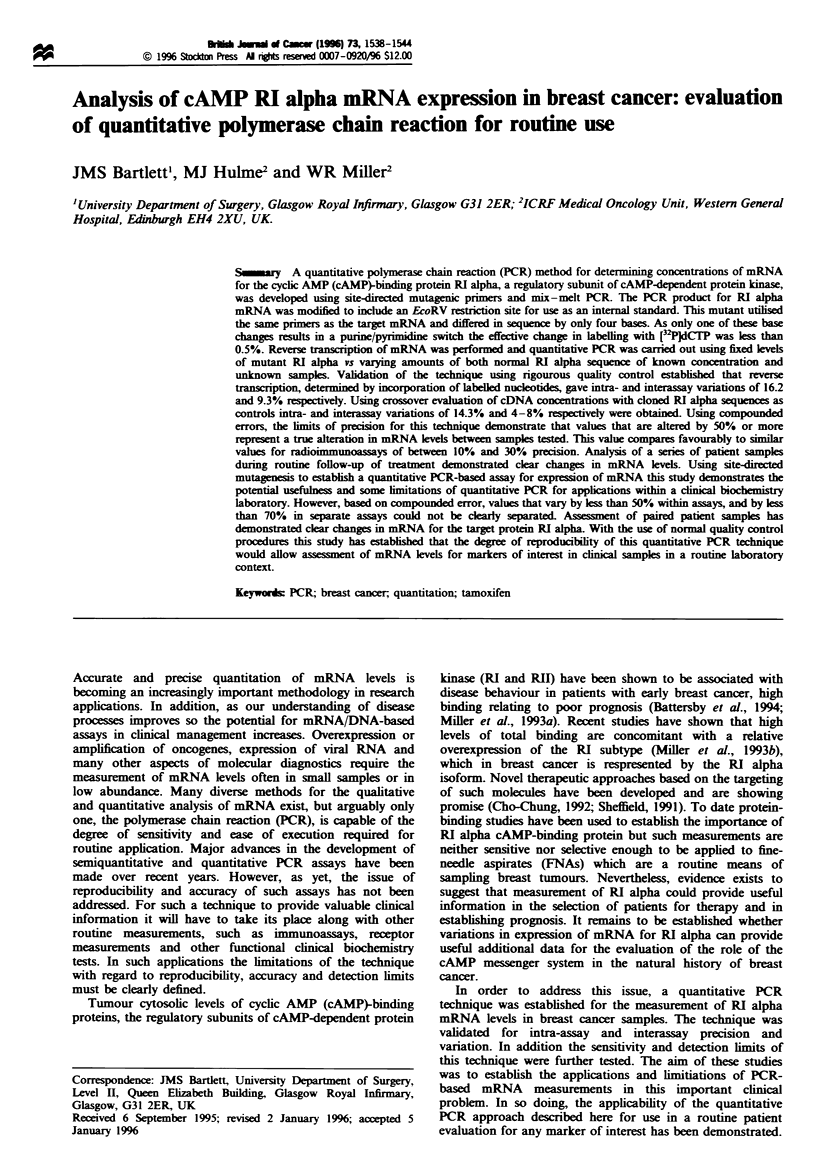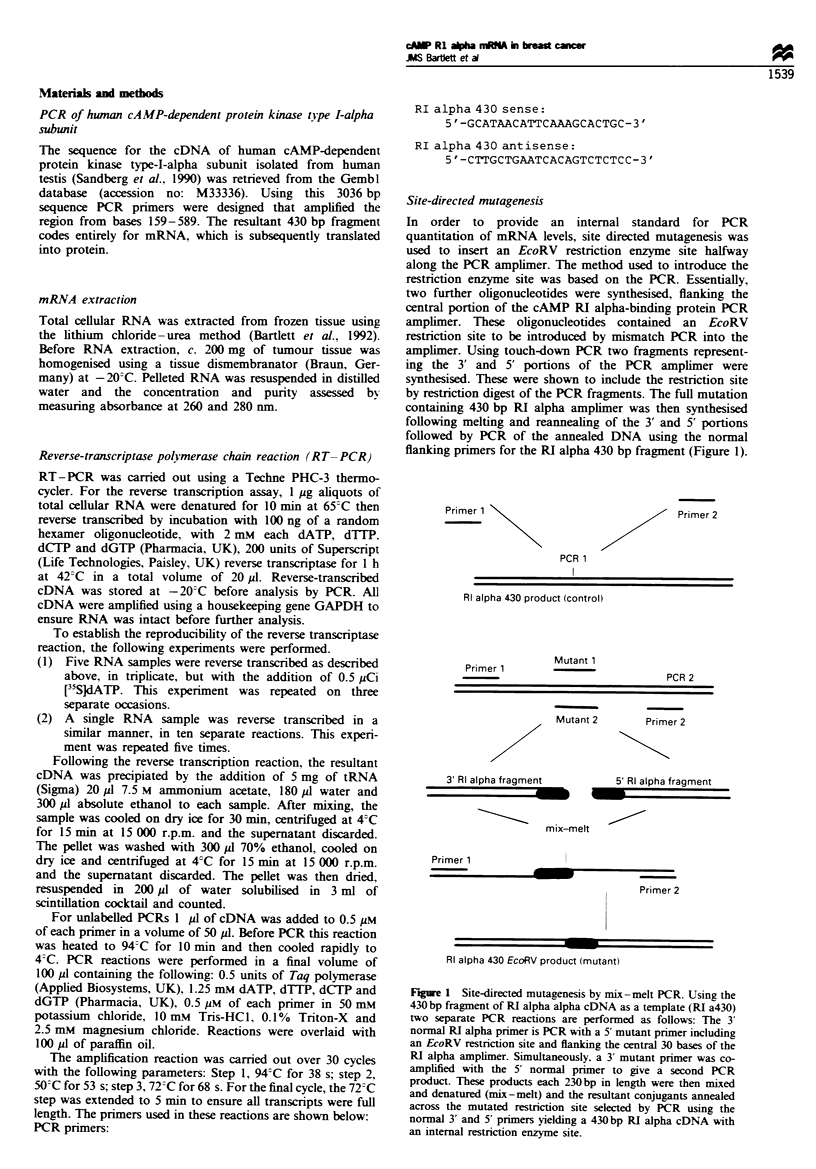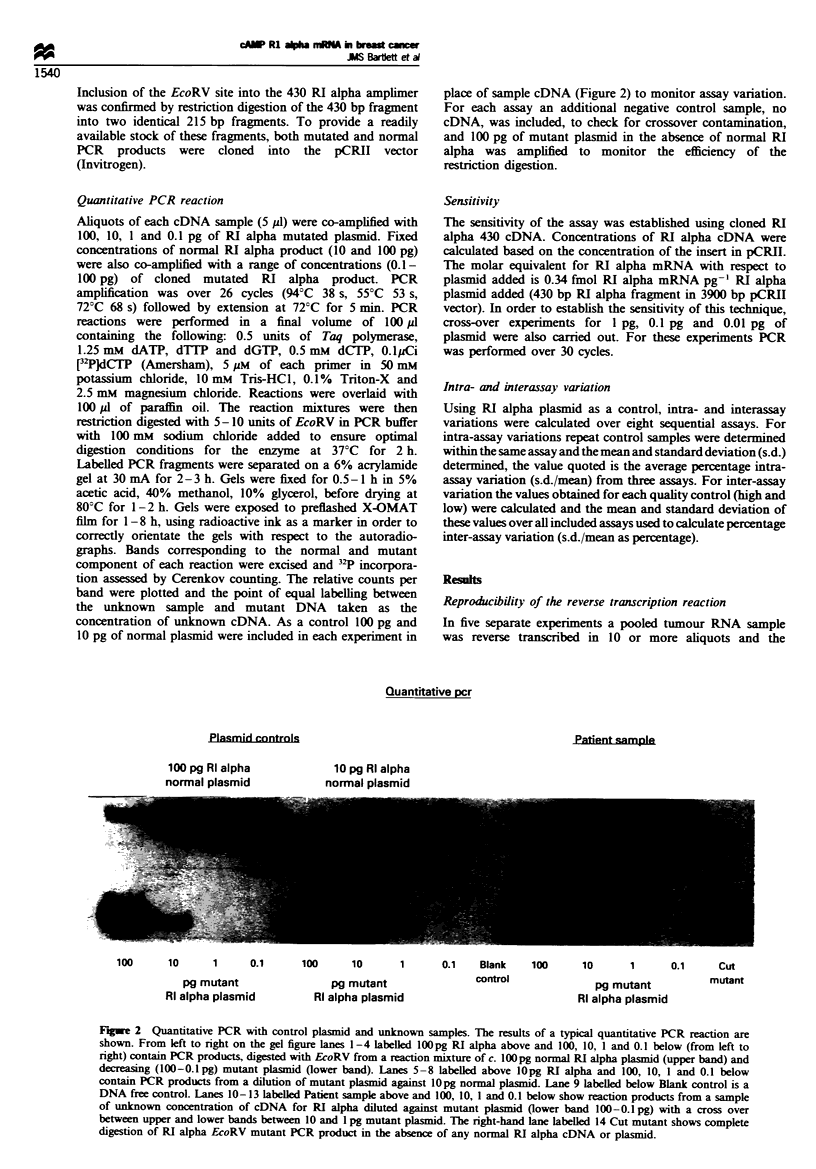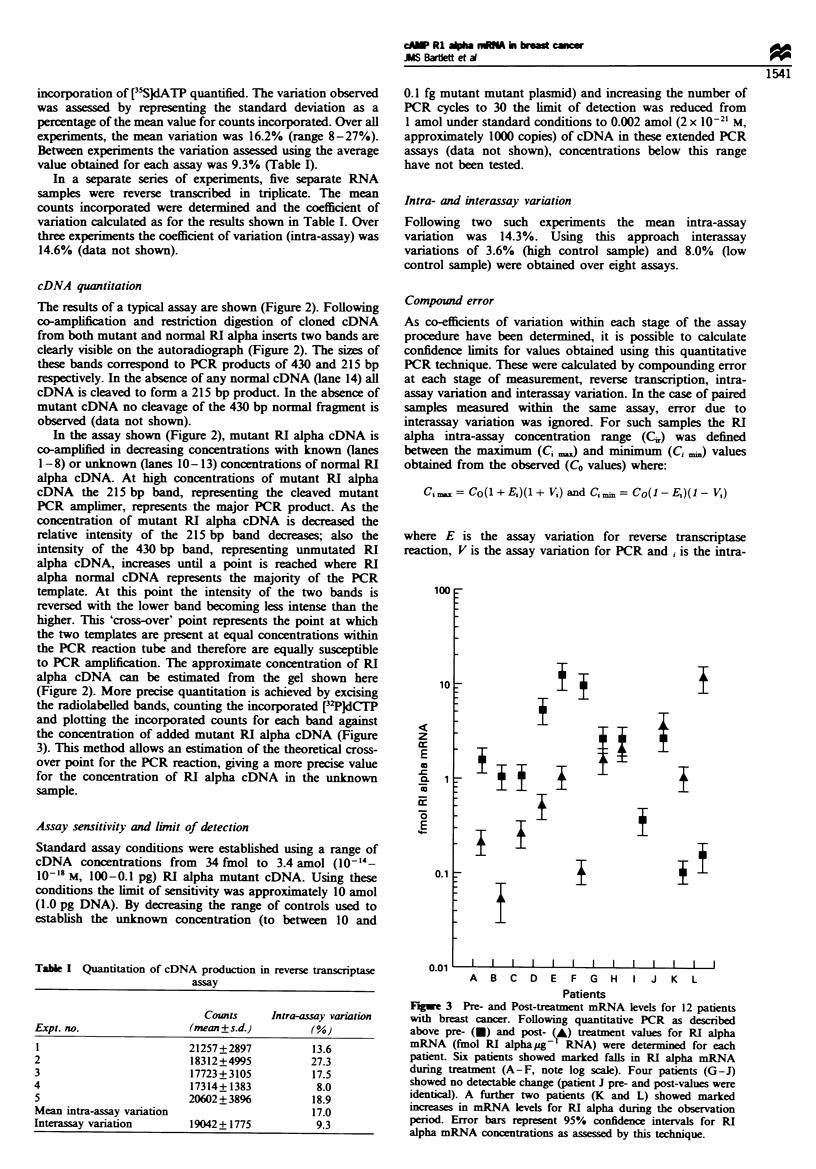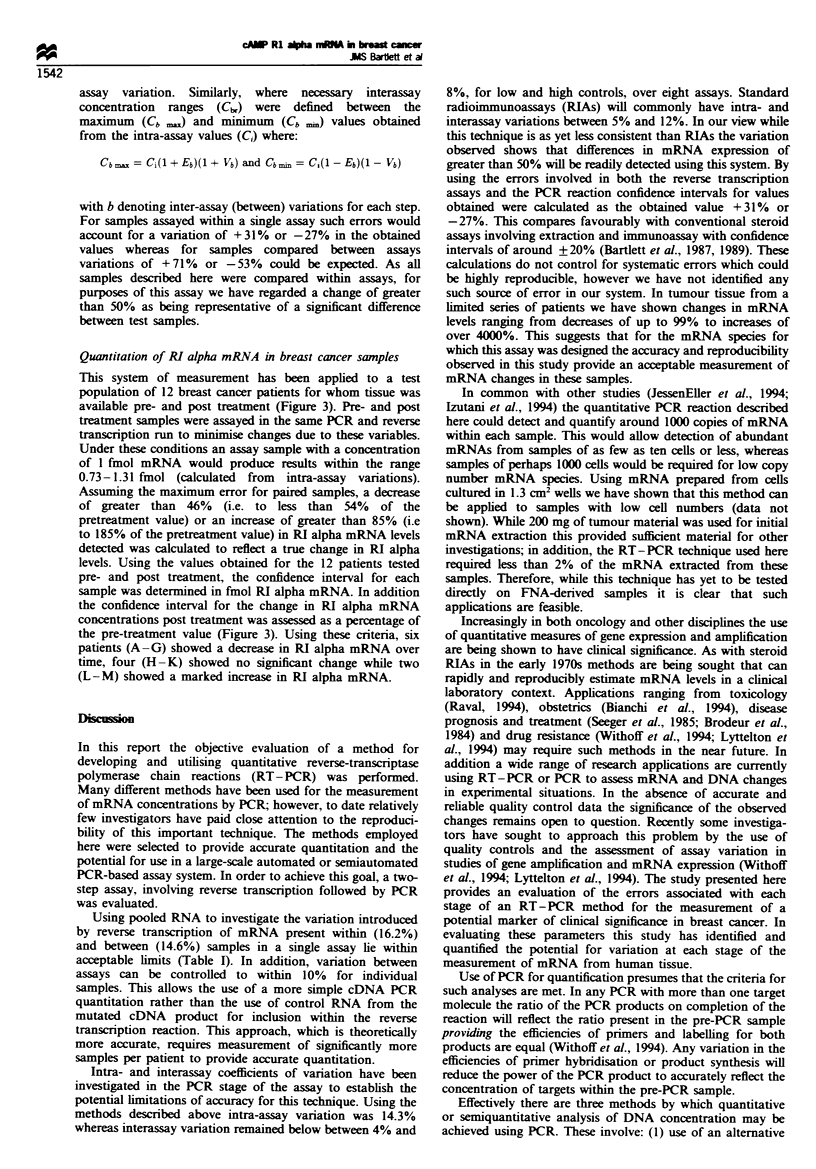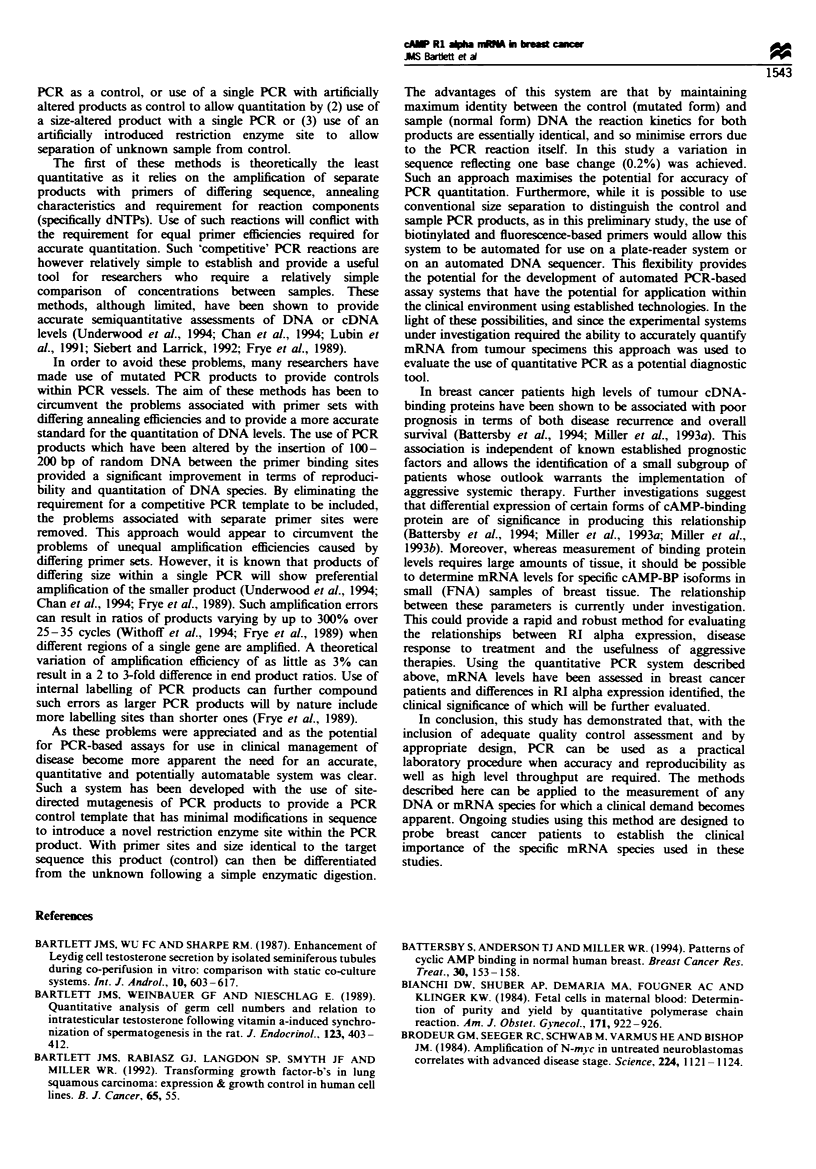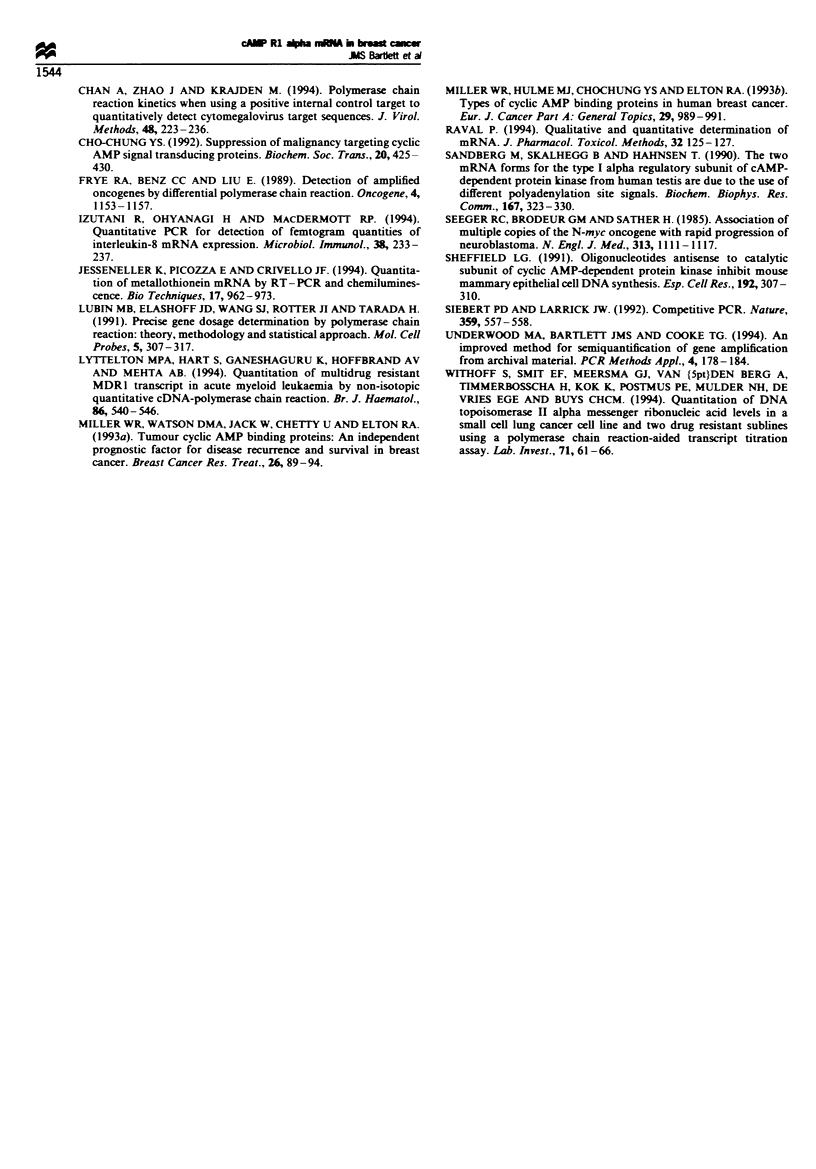# Analysis of cAMP RI alpha mRNA expression in breast cancer: evaluation of quantitative polymerase chain reaction for routine use.

**DOI:** 10.1038/bjc.1996.290

**Published:** 1996-06

**Authors:** J. M. Bartlett, M. J. Hulme, W. R. Miller

**Affiliations:** University Department of Surgery, Glasgow Royal Infirmary, UK.

## Abstract

**Images:**


British Journal of Cancer (1996) 73, 1538-1544

© 1996 Stockton Press All rights reserved 0007-0920/96 $12.00

### Analysis of cAMP RI alpha mRNA expression in breast cancer: evaluation of quantitative polymerase chain reaction for routine use

JMS Bartlett[1], MJ Hulme[2] and WR Miller[2]

[1]*University Department of Surgery, Glasgow Royal Infirmary, Glasgow G31 2ER;* [2]*ICRF Medical Oncology Unit, Western General Hospital, Edinburgh EH4 2XU, UK.*

**Summary** A quantitative polymerase chain reaction (PCR) method for determining concentrations of mRNA for the cyclic AMP (cAMP)-binding protein RI alpha, a regulatory subunit of cAMP-dependent protein kinase, was developed using site-directed mutagenic primers and mix-melt PCR. The PCR product for RI alpha mRNA was modified to include an *Eco*RV restriction site for use as an internal standard. This mutant utilised the same primers as the target mRNA and differed in sequence by only four bases. As only one of these base changes results in a purine/pyrimidine switch the effective change in labelling with [$^{32}$P]dCTP was less than 0.5%. Reverse transcription of mRNA was performed and quantitative PCR was carried out using fixed levels of mutant RI alpha *vs* varying amounts of both normal RI alpha sequence of known concentration and unknown samples. Validation of the technique using rigourous quality control established that reverse transcription, determined by incorporation of labelled nucleotides, gave intra- and interassay variations of 16.2 and 9.3% respectively. Using crossover evaluation of cDNA concentrations with cloned RI alpha sequences as controls intra- and interassay variations of 14.3% and 4–8% respectively were obtained. Using compounded errors, the limits of precision for this technique demonstrate that values that are altered by 50% or more represent a true alteration in mRNA levels between samples tested. This value compares favourably to similar values for radioimmunoassays of between 10% and 30% precision. Analysis of a series of patient samples during routine follow-up of treatment demonstrated clear changes in mRNA levels. Using site-directed mutagenesis to establish a quantitative PCR-based assay for expression of mRNA this study demonstrates the potential usefulness and some limitations of quantitative PCR for applications within a clinical biochemistry laboratory. However, based on compounded error, values that vary by less than 50% within assays, and by less than 70% in separate assays could not be clearly separated. Assessment of paired patient samples has demonstrated clear changes in mRNA for the target protein RI alpha. With the use of normal quality control procedures this study has established that the degree of reproducibility of this quantitative PCR technique would allow assessment of mRNA levels for markers of interest in clinical samples in a routine laboratory context.

**Keywords:** PCR; breast cancer; quantitation; tamoxifen

Accurate and precise quantitation of mRNA levels is becoming an increasingly important methodology in research applications. In addition, as our understanding of disease processes improves so the potential for mRNA/DNA-based assays in clinical management increases. Overexpression or amplification of oncogenes, expression of viral RNA and many other aspects of molecular diagnostics require the measurement of mRNA levels often in small samples or in low abundance. Many diverse methods for the qualitative and quantitative analysis of mRNA exist, but arguably only one, the polymerase chain reaction (PCR), is capable of the degree of sensitivity and ease of execution required for routine application. Major advances in the development of semiquantitative and quantitative PCR assays have been made over recent years. However, as yet, the issue of reproducibility and accuracy of such assays has not been addressed. For such a technique to provide valuable clinical information it will have to take its place along with other routine measurements, such as immunoassays, receptor measurements and other functional clinical biochemistry tests. In such applications the limitations of the technique with regard to reproducibility, accuracy and detection limits must be clearly defined.

Tumour cytosolic levels of cyclic AMP (cAMP)-binding proteins, the regulatory subunits of cAMP-dependent protein kinase (RI and RII) have been shown to be associated with disease behaviour in patients with early breast cancer, high binding relating to poor prognosis (Battersby *et al.*, 1994; Miller *et al.*, 1993*a*). Recent studies have shown that high levels of total binding are concomitant with a relative overexpression of the RI subtype (Miller *et al.*, 1993*b*), which in breast cancer is respresented by the RI alpha isoform. Novel therapeutic approaches based on the targeting of such molecules have been developed and are showing promise (Cho-Chung, 1992; Sheffield, 1991). To date protein-binding studies have been used to establish the importance of RI alpha cAMP-binding protein but such measurements are neither sensitive nor selective enough to be applied to fine-needle aspirates (FNAs) which are a routine means of sampling breast tumours. Nevertheless, evidence exists to suggest that measurement of RI alpha could provide useful information in the selection of patients for therapy and in establishing prognosis. It remains to be established whether variations in expression of mRNA for RI alpha can provide useful additional data for the evaluation of the role of the cAMP messenger system in the natural history of breast cancer.

In order to address this issue, a quantitative PCR technique was established for the measurement of RI alpha mRNA levels in breast cancer samples. The technique was validated for intra-assay and interassay precision and variation. In addition the sensitivity and detection limits of this technique were further tested. The aim of these studies was to establish the applications and limitiations of PCR-based mRNA measurements in this important clinical problem. In so doing, the applicability of the quantitative PCR approach described here for use in a routine patient evaluation for any marker of interest has been demonstrated.

Correspondence: JMS Bartlett, University Department of Surgery, Level II, Queen Elizabeth Building, Glasgow Royal Infirmary, Glasgow, G31 2ER, UK

Received 6 September 1995; revised 2 January 1996; accepted 5 January 1996

### Materials and methods

*PCR of human cAMP-dependent protein kinase type I-alpha subunit*

The sequence for the cDNA of human cAMP-dependent protein kinase type-I-alpha subunit isolated from human testis (Sandberg *et al.*, 1990) was retrieved from the Gembl database (accession no: M33336). Using this 3036 bp sequence PCR primers were designed that amplified the region from bases 159–589. The resultant 430 bp fragment codes entirely for mRNA, which is subsequently translated into protein.

*mRNA extraction*

Total cellular RNA was extracted from frozen tissue using the lithium chloride–urea method (Bartlett *et al.*, 1992). Before RNA extraction, *c.* 200 mg of tumour tissue was homogenised using a tissue dismembranator (Braun, Germany) at −20°C. Pelleted RNA was resuspended in distilled water and the concentration and purity assessed by measuring absorbance at 260 and 280 nm.

*Reverse-transcriptase polymerase chain reaction (RT–PCR)*

RT–PCR was carried out using a Techne PHC-3 thermocycler. For the reverse transcription assay, 1 μg aliquots of total cellular RNA were denatured for 10 min at 65°C then reverse transcribed by incubation with 100 ng of a random hexamer oligonucleotide, with 2 mM each dATP, dTTP, dCTP and dGTP (Pharmacia, UK), 200 units of Superscript (Life Technologies, Paisley, UK) reverse transcriptase for 1 h at 42°C in a total volume of 20 μl. Reverse-transcribed cDNA was stored at −20°C before analysis by PCR. All cDNA were amplified using a housekeeping gene GAPDH to ensure RNA was intact before further analysis.

To establish the reproducibility of the reverse transcriptase reaction, the following experiments were performed.

(1) Five RNA samples were reverse transcribed as described above, in triplicate, but with the addition of 0.5 μCi [$^{35}$S]dATP. This experiment was repeated on three separate occasions.
(2) A single RNA sample was reverse transcribed in a similar manner, in ten separate reactions. This experiment was repeated five times.

Following the reverse transcription reaction, the resultant cDNA was precipiated by the addition of 5 mg of tRNA (Sigma) 20 μl 7.5 M ammonium acetate, 180 μl water and 300 μl absolute ethanol to each sample. After mixing, the sample was cooled on dry ice for 30 min, centrifuged at 4°C for 15 min at 15 000 r.p.m. and the supernatant discarded. The pellet was washed with 300 μl 70% ethanol, cooled on dry ice and centrifuged at 4°C for 15 min at 15 000 r.p.m. and the supernatant discarded. The pellet was then dried, resuspended in 200 μl of water solubilised in 3 ml of scintillation cocktail and counted.

For unlabelled PCRs 1 μl of cDNA was added to 0.5 μM of each primer in a volume of 50 μl. Before PCR this reaction was heated to 94°C for 10 min and then cooled rapidly to 4°C. PCR reactions were performed in a final volume of 100 μl containing the following: 0.5 units of *Taq* polymerase (Applied Biosystems, UK), 1.25 mM dATP, dTTP, dCTP and dGTP (Pharmacia, UK), 0.5 μM of each primer in 50 mM potassium chloride, 10 mM Tris-HCl, 0.1% Triton-X and 2.5 mM magnesium chloride. Reactions were overlaid with 100 μl of paraffin oil.

The amplification reaction was carried out over 30 cycles with the following parameters: Step 1, 94°C for 38 s; step 2, 50°C for 53 s; step 3, 72°C for 68 s. For the final cycle, the 72°C step was extended to 5 min to ensure all transcripts were full length. The primers used in these reactions are shown below: PCR primers:

```
RI alpha 430 sense:
    5'-GCATAACATTCAAAGCACTGC-3'

RI alpha 430 antisense:
    5'-CTTGCTGAATCACAGTCTCTCC-3'
```

*Site-directed mutagenesis*

In order to provide an internal standard for PCR quantitation of mRNA levels, site directed mutagenesis was used to insert an *Eco*RV restriction enzyme site halfway along the PCR amplimer. The method used to introduce the restriction enzyme site was based on the PCR. Essentially, two further oligonucleotides were synthesised, flanking the central portion of the cAMP RI alpha-binding protein PCR amplimer. These oligonucleotides contained an *Eco*RV restriction site to be introduced by mismatch PCR into the amplimer. Using touch-down PCR two fragments representing the 3′ and 5′ portions of the PCR amplimer were synthesised. These were shown to include the restriction site by restriction digest of the PCR fragments. The full mutation containing 430 bp RI alpha amplimer was then synthesised following melting and reannealing of the 3′ and 5′ portions followed by PCR of the annealed DNA using the normal flanking primers for the RI alpha 430 bp fragment (Figure 1).

**Figure 1** Site-directed mutagenesis by mix–melt PCR. Using the 430 bp fragment of RI alpha alpha cDNA as a template (RI a430) two separate PCR reactions are performed as follows: The 3′ normal RI alpha primer is PCR with a 5′ mutant primer including an *Eco*RV restriction site and flanking the central 30 bases of the RI alpha amplimer. Simultaneously, a 3′ mutant primer was co-amplified with the 5′ normal primer to give a second PCR product. These products each 230 bp in length were then mixed and denatured (mix–melt) and the resultant conjugants annealed across the mutated restriction site selected by PCR using the normal 3′ and 5′ primers yielding a 430 bp RI alpha cDNA with an internal restriction enzyme site.

Inclusion of the *Eco*RV site into the 430 RI alpha amplimer was confirmed by restriction digestion of the 430 bp fragment into two identical 215 bp fragments. To provide a readily available stock of these fragments, both mutated and normal PCR products were cloned into the pCRII vector (Invitrogen).

*Quantitative PCR reaction*

Aliquots of each cDNA sample (5 $\mu$l) were co-amplified with 100, 10, 1 and 0.1 pg of RI alpha mutated plasmid. Fixed concentrations of normal RI alpha product (10 and 100 pg) were also co-amplified with a range of concentrations (0.1–100 pg) of cloned mutated RI alpha product. PCR amplification was over 26 cycles (94°C 38 s, 55°C 53 s, 72°C 68 s) followed by extension at 72°C for 5 min. PCR reactions were performed in a final volume of 100 $\mu$l containing the following: 0.5 units of *Taq* polymerase, 1.25 mM dATP, dTTP and dGTP, 0.5 mM dCTP, 0.1$\mu$Ci [$^{32}$P]dCTP (Amersham), 5 $\mu$M of each primer in 50 mM potassium chloride, 10 mM Tris-HC1, 0.1% Triton-X and 2.5 mM magnesium chloride. Reactions were overlaid with 100 $\mu$l of paraffin oil. The reaction mixtures were then restriction digested with 5–10 units of *Eco*RV in PCR buffer with 100 mM sodium chloride added to ensure optimal digestion conditions for the enzyme at 37°C for 2 h. Labelled PCR fragments were separated on a 6% acrylamide gel at 30 mA for 2–3 h. Gels were fixed for 0.5–1 h in 5% acetic acid, 40% methanol, 10% glycerol, before drying at 80°C for 1–2 h. Gels were exposed to preflashed X-OMAT film for 1–8 h, using radioactive ink as a marker in order to correctly orientate the gels with respect to the autoradiographs. Bands corresponding to the normal and mutant component of each reaction were excised and $^{32}$P incorporation assessed by Cerenkov counting. The relative counts per band were plotted and the point of equal labelling between the unknown sample and mutant DNA taken as the concentration of unknown cDNA. As a control 100 pg and 10 pg of normal plasmid were included in each experiment in place of sample cDNA (Figure 2) to monitor assay variation. For each assay an additional negative control sample, no cDNA, was included, to check for crossover contamination, and 100 pg of mutant plasmid in the absence of normal RI alpha was amplified to monitor the efficiency of the restriction digestion.

*Sensitivity*

The sensitivity of the assay was established using cloned RI alpha 430 cDNA. Concentrations of RI alpha cDNA were calculated based on the concentration of the insert in pCRII. The molar equivalent for RI alpha mRNA with respect to plasmid added is 0.34 fmol RI alpha mRNA pg$^{-1}$ RI alpha plasmid added (430 bp RI alpha fragment in 3900 bp pCRII vector). In order to establish the sensitivity of this technique, cross-over experiments for 1 pg, 0.1 pg and 0.01 pg of plasmid were also carried out. For these experiments PCR was performed over 30 cycles.

*Intra- and interassay variation*

Using RI alpha plasmid as a control, intra- and interassay variations were calculated over eight sequential assays. For intra-assay variations repeat control samples were determined within the same assay and the mean and standard deviation (s.d.) determined, the value quoted is the average percentage intra-assay variation (s.d./mean) from three assays. For inter-assay variation the values obtained for each quality control (high and low) were calculated and the mean and standard deviation of these values over all included assays used to calculate percentage inter-assay variation (s.d./mean as percentage).

### Results

*Reproducibility of the reverse transcription reaction*

In five separate experiments a pooled tumour RNA sample was reverse transcribed in 10 or more aliquots and the

**Figure 2** Quantitative PCR with control plasmid and unknown samples. The results of a typical quantitative PCR reaction are shown. From left to right on the gel figure lanes 1–4 labelled 100 pg RI alpha above and 100, 10, 1 and 0.1 below (from left to right) contain PCR products, digested with *Eco*RV from a reaction mixture of *c*. 100 pg normal RI alpha plasmid (upper band) and decreasing (100–0.1 pg) mutant plasmid (lower band). Lanes 5–8 labelled above 10 pg RI alpha and 100, 10, 1 and 0.1 below contain PCR products from a dilution of mutant plasmid against 10 pg normal plasmid. Lane 9 labelled below Blank control is a DNA free control. Lanes 10–13 labelled Patient sample above and 100, 10, 1 and 0.1 below show reaction products from a sample of unknown concentration of cDNA for RI alpha diluted against mutant plasmid (lower band 100–0.1 pg) with a cross over between upper and lower bands between 10 and 1 pg mutant plasmid. The right-hand lane labelled 14 Cut mutant shows complete digestion of RI alpha *Eco*RV mutant PCR product in the absence of any normal RI alpha cDNA or plasmid.

incorporation of [$^{35}$S]dATP quantified. The variation observed was assessed by representing the standard deviation as a percentage of the mean value for counts incorporated. Over all experiments, the mean variation was 16.2% (range 8–27%). Between experiments the variation assessed using the average value obtained for each assay was 9.3% (Table I).

In a separate series of experiments, five separate RNA samples were reverse transcribed in triplicate. The mean counts incorporated were determined and the coefficient of variation calculated as for the results shown in Table I. Over three experiments the coefficient of variation (intra-assay) was 14.6% (data not shown).

### *cDNA quantitation*

The results of a typical assay are shown (Figure 2). Following co-amplification and restriction digestion of cloned cDNA from both mutant and normal RI alpha inserts two bands are clearly visible on the autoradiograph (Figure 2). The sizes of these bands correspond to PCR products of 430 and 215 bp respectively. In the absence of any normal cDNA (lane 14) all cDNA is cleaved to form a 215 bp product. In the absence of mutant cDNA no cleavage of the 430 bp normal fragment is observed (data not shown).

In the assay shown (Figure 2), mutant RI alpha cDNA is co-amplified in decreasing concentrations with known (lanes 1–8) or unknown (lanes 10–13) concentrations of normal RI alpha cDNA. At high concentrations of mutant RI alpha cDNA the 215 bp band, representing the cleaved mutant PCR amplimer, represents the major PCR product. As the concentration of mutant RI alpha cDNA is decreased the relative intensity of the 215 bp band decreases; also the intensity of the 430 bp band, representing unmutated RI alpha cDNA, increases until a point is reached where RI alpha normal cDNA represents the majority of the PCR template. At this point the intensity of the two bands is reversed with the lower band becoming less intense than the higher. This 'cross-over' point represents the point at which the two templates are present at equal concentrations within the PCR reaction tube and therefore are equally susceptible to PCR amplification. The approximate concentration of RI alpha cDNA can be estimated from the gel shown here (Figure 2). More precise quantitation is achieved by excising the radiolabelled bands, counting the incorporated [$^{32}$P]dCTP and plotting the incorporated counts for each band against the concentration of added mutant RI alpha cDNA (Figure 3). This method allows an estimation of the theoretical cross-over point for the PCR reaction, giving a more precise value for the concentration of RI alpha cDNA in the unknown sample.

### *Assay sensitivity and limit of detection*

Standard assay conditions were established using a range of cDNA concentrations from 34 fmol to 3.4 amol ($10^{-14}$–$10^{-18}$ M, 100–0.1 pg) RI alpha mutant cDNA. Using these conditions the limit of sensitivity was approximately 10 amol (1.0 pg DNA). By decreasing the range of controls used to establish the unknown concentration (to between 10 and

**Table I** Quantitation of cDNA production in reverse transcriptase assay

| *Expt. no.* | *Counts (mean ± s.d.)* | *Intra-assay variation (%)* |
|---|---|---|
| 1 | 21257±2897 | 13.6 |
| 2 | 18312±4995 | 27.3 |
| 3 | 17723±3105 | 17.5 |
| 4 | 17314±1383 | 8.0 |
| 5 | 20602±3896 | 18.9 |
| Mean intra-assay variation | | 17.0 |
| Interassay variation | 19042±1775 | 9.3 |

0.1 fg mutant mutant plasmid) and increasing the number of PCR cycles to 30 the limit of detection was reduced from 1 amol under standard conditions to 0.002 amol ($2 \times 10^{-21}$ M, approximately 1000 copies) of cDNA in these extended PCR assays (data not shown), concentrations below this range have not been tested.

### *Intra- and interassay variation*

Following two such experiments the mean intra-assay variation was 14.3%. Using this approach interassay variations of 3.6% (high control sample) and 8.0% (low control sample) were obtained over eight assays.

### *Compound error*

As co-efficients of variation within each stage of the assay procedure have been determined, it is possible to calculate confidence limits for values obtained using this quantitative PCR technique. These were calculated by compounding error at each stage of measurement, reverse transcription, intra-assay variation and interassay variation. In the case of paired samples measured within the same assay, error due to interassay variation was ignored. For such samples the RI alpha intra-assay concentration range ($C_{ir}$) was defined between the maximum ($C_{i\,max}$) and minimum ($C_{i\,min}$) values obtained from the observed ($C_0$ values) where:

$$C_{i\,max} = C_O(1 + E_i)(1 + V_i) \text{ and } C_{i\,min} = C_O(1 - E_i)(1 - V_i)$$

where $E$ is the assay variation for reverse transcriptase reaction, $V$ is the assay variation for PCR and $_i$ is the intra-

**Figure 3** Pre- and Post-treatment mRNA levels for 12 patients with breast cancer. Following quantitative PCR as described above pre- (■) and post- (▲) treatment values for RI alpha mRNA (fmol RI alpha $\mu g^{-1}$ RNA) were determined for each patient. Six patients showed marked falls in RI alpha mRNA during treatment (A–F, note log scale). Four patients (G–J) showed no detectable change (patient J pre- and post-values were identical). A further two patients (K and L) showed marked increases in mRNA levels for RI alpha during the observation period. Error bars represent 95% confidence intervals for RI alpha mRNA concentrations as assessed by this technique.

assay variation. Similarly, where necessary interassay concentration ranges ($C_{br}$) were defined between the maximum ($C_{b\ max}$) and minimum ($C_{b\ min}$) values obtained from the intra-assay values ($C_i$) where:

$$C_{b\,max} = C_i(1 + E_b)(1 + V_b) \text{ and } C_{b\,min} = C_i(1 - E_b)(1 - V_b)$$

with $b$ denoting inter-assay (between) variations for each step. For samples assayed within a single assay such errors would account for a variation of +31% or −27% in the obtained values whereas for samples compared between assays variations of +71% or −53% could be expected. As all samples described here were compared within assays, for purposes of this assay we have regarded a change of greater than 50% as being representative of a significant difference between test samples.

*Quantitation of RI alpha mRNA in breast cancer samples*

This system of measurement has been applied to a test population of 12 breast cancer patients for whom tissue was available pre- and post treatment (Figure 3). Pre- and post treatment samples were assayed in the same PCR and reverse transcription run to minimise changes due to these variables. Under these conditions an assay sample with a concentration of 1 fmol mRNA would produce results within the range 0.73–1.31 fmol (calculated from intra-assay variations). Assuming the maximum error for paired samples, a decrease of greater than 46% (i.e. to less than 54% of the pretreatment value) or an increase of greater than 85% (i.e to 185% of the pretreatment value) in RI alpha mRNA levels detected was calculated to reflect a true change in RI alpha levels. Using the values obtained for the 12 patients tested pre- and post treatment, the confidence interval for each sample was determined in fmol RI alpha mRNA. In addition the confidence interval for the change in RI alpha mRNA concentrations post treatment was assessed as a percentage of the pre-treatment value (Figure 3). Using these criteria, six patients (A–G) showed a decrease in RI alpha mRNA over time, four (H–K) showed no significant change while two (L–M) showed a marked increase in RI alpha mRNA.

### Discussion

In this report the objective evaluation of a method for developing and utilising quantitative reverse-transcriptase polymerase chain reactions (RT–PCR) was performed. Many different methods have been used for the measurement of mRNA concentrations by PCR; however, to date relatively few investigators have paid close attention to the reproducibility of this important technique. The methods employed here were selected to provide accurate quantitation and the potential for use in a large-scale automated or semiautomated PCR-based assay system. In order to achieve this goal, a two-step assay, involving reverse transcription followed by PCR was evaluated.

Using pooled RNA to investigate the variation introduced by reverse transcription of mRNA present within (16.2%) and between (14.6%) samples in a single assay lie within acceptable limits (Table I). In addition, variation between assays can be controlled to within 10% for individual samples. This allows the use of a more simple cDNA PCR quantitation rather than the use of control RNA from the mutated cDNA product for inclusion within the reverse transcription reaction. This approach, which is theoretically more accurate, requires measurement of significantly more samples per patient to provide accurate quantitation.

Intra- and interassay coefficients of variation have been investigated in the PCR stage of the assay to establish the potential limitations of accuracy for this technique. Using the methods described above intra-assay variation was 14.3% whereas interassay variation remained below between 4% and 8%, for low and high controls, over eight assays. Standard radioimmunoassays (RIAs) will commonly have intra- and interassay variations between 5% and 12%. In our view while this technique is as yet less consistent than RIAs the variation observed shows that differences in mRNA expression of greater than 50% will be readily detected using this system. By using the errors involved in both the reverse transcription assays and the PCR reaction confidence intervals for values obtained were calculated as the obtained value +31% or −27%. This compares favourably with conventional steroid assays involving extraction and immunoassay with confidence intervals of around ±20% (Bartlett *et al.*, 1987, 1989). These calculations do not control for systematic errors which could be highly reproducible, however we have not identified any such source of error in our system. In tumour tissue from a limited series of patients we have shown changes in mRNA levels ranging from decreases of up to 99% to increases of over 4000%. This suggests that for the mRNA species for which this assay was designed the accuracy and reproducibility observed in this study provide an acceptable measurement of mRNA changes in these samples.

In common with other studies (JessenEller *et al.*, 1994; Izutani *et al.*, 1994) the quantitative PCR reaction described here could detect and quantify around 1000 copies of mRNA within each sample. This would allow detection of abundant mRNAs from samples of as few as ten cells or less, whereas samples of perhaps 1000 cells would be required for low copy number mRNA species. Using mRNA prepared from cells cultured in 1.3 $cm^2$ wells we have shown that this method can be applied to samples with low cell numbers (data not shown). While 200 mg of tumour material was used for initial mRNA extraction this provided sufficient material for other investigations; in addition, the RT–PCR technique used here required less than 2% of the mRNA extracted from these samples. Therefore, while this technique has yet to be tested directly on FNA-derived samples it is clear that such applications are feasible.

Increasingly in both oncology and other disciplines the use of quantitative measures of gene expression and amplification are being shown to have clinical significance. As with steroid RIAs in the early 1970s methods are being sought that can rapidly and reproducibly estimate mRNA levels in a clinical laboratory context. Applications ranging from toxicology (Raval, 1994), obstetrics (Bianchi *et al.*, 1994), disease prognosis and treatment (Seeger *et al.*, 1985; Brodeur *et al.*, 1984) and drug resistance (Withoff *et al.*, 1994; Lyttelton *et al.*, 1994) may require such methods in the near future. In addition a wide range of research applications are currently using RT–PCR or PCR to assess mRNA and DNA changes in experimental situations. In the absence of accurate and reliable quality control data the significance of the observed changes remains open to question. Recently some investigators have sought to approach this problem by the use of quality controls and the assessment of assay variation in studies of gene amplification and mRNA expression (Withoff *et al.*, 1994; Lyttelton *et al.*, 1994). The study presented here provides an evaluation of the errors associated with each stage of an RT–PCR method for the measurement of a potential marker of clinical significance in breast cancer. In evaluating these parameters this study has identified and quantified the potential for variation at each stage of the measurement of mRNA from human tissue.

Use of PCR for quantification presumes that the criteria for such analyses are met. In any PCR with more than one target molecule the ratio of the PCR products on completion of the reaction will reflect the ratio present in the pre-PCR sample *providing* the efficiencies of primers and labelling for both products are equal (Withoff *et al.*, 1994). Any variation in the efficiencies of primer hybridisation or product synthesis will reduce the power of the PCR product to accurately reflect the concentration of targets within the pre-PCR sample.

Effectively there are three methods by which quantitative or semiquantitative analysis of DNA concentration may be achieved using PCR. These involve: (1) use of an alternative

PCR as a control, or use of a single PCR with artificially altered products as control to allow quantitation by (2) use of a size-altered product with a single PCR or (3) use of an artificially introduced restriction enzyme site to allow separation of unknown sample from control.

The first of these methods is theoretically the least quantitative as it relies on the amplification of separate products with primers of differing sequence, annealing characteristics and requirement for reaction components (specifically dNTPs). Use of such reactions will conflict with the requirement for equal primer efficiencies required for accurate quantitation. Such 'competitive' PCR reactions are however relatively simple to establish and provide a useful tool for researchers who require a relatively simple comparison of concentrations between samples. These methods, although limited, have been shown to provide accurate semiquantitative assessments of DNA or cDNA levels (Underwood *et al.*, 1994; Chan *et al.*, 1994; Lubin *et al.*, 1991; Siebert and Larrick, 1992; Frye *et al.*, 1989).

In order to avoid these problems, many researchers have made use of mutated PCR products to provide controls within PCR vessels. The aim of these methods has been to circumvent the problems associated with primer sets with differing annealing efficiencies and to provide a more accurate standard for the quantitation of DNA levels. The use of PCR products which have been altered by the insertion of 100–200 bp of random DNA between the primer binding sites provided a significant improvement in terms of reproducibility and quantitation of DNA species. By eliminating the requirement for a competitive PCR template to be included, the problems associated with separate primer sites were removed. This approach would appear to circumvent the problems of unequal amplification efficiencies caused by differing primer sets. However, it is known that products of differing size within a single PCR will show preferential amplification of the smaller product (Underwood *et al.*, 1994; Chan *et al.*, 1994; Frye *et al.*, 1989). Such amplification errors can result in ratios of products varying by up to 300% over 25–35 cycles (Withoff *et al.*, 1994; Frye *et al.*, 1989) when different regions of a single gene are amplified. A theoretical variation of amplification efficiency of as little as 3% can result in a 2 to 3-fold difference in end product ratios. Use of internal labelling of PCR products can further compound such errors as larger PCR products will by nature include more labelling sites than shorter ones (Frye *et al.*, 1989).

As these problems were appreciated and as the potential for PCR-based assays for use in clinical management of disease become more apparent the need for an accurate, quantitative and potentially automatable system was clear. Such a system has been developed with the use of site-directed mutagenesis of PCR products to provide a PCR control template that has minimal modifications in sequence to introduce a novel restriction enzyme site within the PCR product. With primer sites and size identical to the target sequence this product (control) can then be differentiated from the unknown following a simple enzymatic digestion. The advantages of this system are that by maintaining maximum identity between the control (mutated form) and sample (normal form) DNA the reaction kinetics for both products are essentially identical, and so minimise errors due to the PCR reaction itself. In this study a variation in sequence reflecting one base change (0.2%) was achieved. Such an approach maximises the potential for accuracy of PCR quantitation. Furthermore, while it is possible to use conventional size separation to distinguish the control and sample PCR products, as in this preliminary study, the use of biotinylated and fluorescence-based primers would allow this system to be automated for use on a plate-reader system or on an automated DNA sequencer. This flexibility provides the potential for the development of automated PCR-based assay systems that have the potential for application within the clinical environment using established technologies. In the light of these possibilities, and since the experimental systems under investigation required the ability to accurately quantify mRNA from tumour specimens this approach was used to evaluate the use of quantitative PCR as a potential diagnostic tool.

In breast cancer patients high levels of tumour cDNA-binding proteins have been shown to be associated with poor prognosis in terms of both disease recurrence and overall survival (Battersby *et al.*, 1994; Miller *et al.*, 1993*a*). This association is independent of known established prognostic factors and allows the identification of a small subgroup of patients whose outlook warrants the implementation of aggressive systemic therapy. Further investigations suggest that differential expression of certain forms of cAMP-binding protein are of significance in producing this relationship (Battersby *et al.*, 1994; Miller *et al.*, 1993*a*; Miller *et al.*, 1993*b*). Moreover, whereas measurement of binding protein levels requires large amounts of tissue, it should be possible to determine mRNA levels for specific cAMP-BP isoforms in small (FNA) samples of breast tissue. The relationship between these parameters is currently under investigation. This could provide a rapid and robust method for evaluating the relationships between RI alpha expression, disease response to treatment and the usefulness of aggressive therapies. Using the quantitative PCR system described above, mRNA levels have been assessed in breast cancer patients and differences in RI alpha expression identified, the clinical significance of which will be further evaluated.

In conclusion, this study has demonstrated that, with the inclusion of adequate quality control assessment and by appropriate design, PCR can be used as a practical laboratory procedure when accuracy and reproducibility as well as high level throughput are required. The methods described here can be applied to the measurement of any DNA or mRNA species for which a clinical demand becomes apparent. Ongoing studies using this method are designed to probe breast cancer patients to establish the clinical importance of the specific mRNA species used in these studies.

### References

BARTLETT JMS, WU FC AND SHARPE RM. (1987). Enhancement of Leydig cell testosterone secretion by isolated seminiferous tubules during co-perifusion in vitro: comparison with static co-culture systems. *Int. J. Androl.*, **10,** 603–617.

BARTLETT JMS, WEINBAUER GF AND NIESCHLAG E. (1989). Quantitative analysis of germ cell numbers and relation to intratesticular testosterone following vitamin a-induced synchronization of spermatogenesis in the rat. *J. Endocrinol.*, **123,** 403–412.

BARTLETT JMS, RABIASZ GJ, LANGDON SP, SMYTH JF AND MILLER WR. (1992). Transforming growth factor-b's in lung squamous carcinoma: expression & growth control in human cell lines. *B. J. Cancer*, **65,** 55.

BATTERSBY S, ANDERSON TJ AND MILLER WR. (1994). Patterns of cyclic AMP binding in normal human breast. *Breast Cancer Res. Treat.*, **30,** 153–158.

BIANCHI DW, SHUBER AP, DEMARIA MA, FOUGNER AC AND KLINGER KW. (1984). Fetal cells in maternal blood: Determination of purity and yield by quantitative polymerase chain reaction. *Am. J. Obstet. Gynecol.*, **171,** 922–926.

BRODEUR GM, SEEGER RC, SCHWAB M, VARMUS HE AND BISHOP JM. (1984). Amplification of N-*myc* in untreated neuroblastomas correlates with advanced disease stage. *Science*, **224,** 1121–1124.

CHAN A, ZHAO J AND KRAJDEN M. (1994). Polymerase chain reaction kinetics when using a positive internal control target to quantitatively detect cytomegalovirus target sequences. *J. Virol. Methods*, **48**, 223-236.

CHO-CHUNG YS. (1992). Suppression of malignancy targeting cyclic AMP signal transducing proteins. *Biochem. Soc. Trans.*, **20**, 425-430.

FRYE RA, BENZ CC AND LIU E. (1989). Detection of amplified oncogenes by differential polymerase chain reaction. *Oncogene*, **4**, 1153-1157.

IZUTANI R, OHYANAGI H AND MACDERMOTT RP. (1994). Quantitative PCR for detection of femtogram quantities of interleukin-8 mRNA expression. *Microbiol. Immunol.*, **38**, 233-237.

JESSENELLER K, PICOZZA E AND CRIVELLO JF. (1994). Quantitation of metallothionein mRNA by RT-PCR and chemiluminescence. *Bio Techniques*, **17**, 962-973.

LUBIN MB, ELASHOFF JD, WANG SJ, ROTTER JI AND TARADA H. (1991). Precise gene dosage determination by polymerase chain reaction: theory, methodology and statistical approach. *Mol. Cell Probes*, **5**, 307-317.

LYTTELTON MPA, HART S, GANESHAGURU K, HOFFBRAND AV AND MEHTA AB. (1994). Quantitation of multidrug resistant MDR1 transcript in acute myeloid leukaemia by non-isotopic quantitative cDNA-polymerase chain reaction. *Br. J. Haematol.*, **86**, 540-546.

MILLER WR, WATSON DMA, JACK W, CHETTY U AND ELTON RA. (1993*a*). Tumour cyclic AMP binding proteins: An independent prognostic factor for disease recurrence and survival in breast cancer. *Breast Cancer Res. Treat.*, **26**, 89-94.

MILLER WR, HULME MJ, CHOCHUNG YS AND ELTON RA. (1993*b*). Types of cyclic AMP binding proteins in human breast cancer. *Eur. J. Cancer Part A: General Topics*, **29**, 989-991.

RAVAL P. (1994). Qualitative and quantitative determination of mRNA. *J. Pharmacol. Toxicol. Methods*, **32** 125-127.

SANDBERG M, SKALHEGG B AND HAHNSEN T. (1990). The two mRNA forms for the type I alpha regulatory subunit of cAMP-dependent protein kinase from human testis are due to the use of different polyadenylation site signals. *Biochem. Biophys. Res. Comm.*, **167**, 323-330.

SEEGER RC, BRODEUR GM AND SATHER H. (1985). Association of multiple copies of the N-*myc* oncogene with rapid progression of neuroblastoma. *N. Engl. J. Med.*, **313**, 1111-1117.

SHEFFIELD LG. (1991). Oligonucleotides antisense to catalytic subunit of cyclic AMP-dependent protein kinase inhibit mouse mammary epithelial cell DNA synthesis. *Esp. Cell Res.*, **192**, 307-310.

SIEBERT PD AND LARRICK JW. (1992). Competitive PCR. *Nature*, **359**, 557-558.

UNDERWOOD MA, BARTLETT JMS AND COOKE TG. (1994). An improved method for semiquantification of gene amplification from archival material. *PCR Methods Appl.*, **4**, 178-184.

WITHOFF S, SMIT EF, MEERSMA GJ, VAN {5pt}DEN BERG A, TIMMERBOSSCHA H, KOK K, POSTMUS PE, MULDER NH, DE VRIES EGE AND BUYS CHCM. (1994). Quantitation of DNA topoisomerase II alpha messenger ribonucleic acid levels in a small cell lung cancer cell line and two drug resistant sublines using a polymerase chain reaction-aided transcript titration assay. *Lab. Invest.*, **71**, 61-66.